# Upper Ureteral Reconstruction with a Tapered Descending Colon after Failed Pyeloplasties in a 13-Year-Old Boy

**DOI:** 10.1055/a-2035-4637

**Published:** 2023-04-10

**Authors:** Hideaki Nakajima, Hiroyuki Koga, Seitaro Kosaka, Mao Ikari, Geoffrey J. Lane, Atsuyuki Yamataka

**Affiliations:** 1 Department of Pediatric General & Urogenital Surgery, Juntendo University School of Medicine Graduate School of Medicine, Bunkyo-ku, Tokyo, Japan; 2 Department of Pediatric Surgery, Juntendo University Shizuoka Hospital, Izunokuni, Shizuoka, Japan

**Keywords:** ureteral reconstruction, ureteropelvic junction obstruction, tapered segment of the descending colon, long ureteral stenosis

## Abstract

An 11-year-old boy was referred for further management of a 6-cm-long grossly stenosed ureter following two failed left ureteropelvic junction (UPJ) obstruction repairs elsewhere. A tapered segment of the descending colon (TDC) was used successfully for ureteral reconstruction. The UPJ was exposed through a left flank incision. The stenosed segment was excised; both ends appeared severely inflamed and thickened. Tissue interposition was required and ureteroplasty with a TDC was performed by incising the peritoneum adjacent to the excised ureter to mobilize the descending colon to the retroperitoneal space. To prepare the TDC, an 8-cm segment of the colon with intact blood vessels was isolated, tapered, and sutured into a funnel shape using a 14-Fr catheter as a temporary stent. After colocolostomy, the colon was returned to the abdominal cavity, the peritoneum was closed carefully to prevent vascular compromise, and the TDC was anastomosed to the ureter and renal calyx with interrupted absorbable sutures. A double J stent (DJS) and percutaneous nephrostomy tube were placed. Postoperative recovery was uneventful. The DJS was removed on day 50 after confirming smooth urine flow through both the ureter–TDC and calyx–TDC anastomoses. Diuretic renography performed 68 days postoperatively was unobstructed. The patient is currently well after 12 months follow-up. This would appear to be the first report of a TDC being used to create a funnel-shaped segment to reconstruct a long, grossly stenosed ureter. The TDC is simpler than the re-tubularizing colon but requires monitoring for postoperative mucus-related complications and malignant transformation.

## Introduction

The surgical management of ureteral stenosis varies according to the level and length of the defect. Short upper ureteral stenosis may be treated by excision and ureteroureterostomy or ureterocalicostomy. Long upper ureteral stenosis requires more extensive intervention such as transureteroureterostomy, renal auto-transplantation, tissue interposition with gastrointestinal tract (GIT) segments or ureteral reconstruction using the ileum, colon, appendix, and other tissue like buccal mucosal grafts, 1 2 3 4 5 6 7 8 or the Yang–Monti technique using transverse re-tubularized segments of the ileum. Of these options, the Yang–Monti technique has been reported to be effective. 8

The colon may have limited application for ureteral reconstruction in its original form because the resulting segment will have a larger diameter and surface area because it is generally wider and thicker than other tissues. However, in the presented case, the descending colon was the most readily available tissue in close proximity to the stenosed ureter and was reconfigured by tapering to create a substitute ureter.

In view of the previous surgical history of the presented case, involving two failed left ureteropelvic junction (UPJ) obstruction repairs that resulted in extensive ureteral stenosis of 5 cm, a tapered segment of the descending colon (TDC) was considered the only viable option for ureteral reconstruction and demonstrated skillful application of existing surgical techniques for treating a unique case of gross ureteral stenosis. The outcome was successful and the patient has been well.

## Case Report

**Fig. 1:**
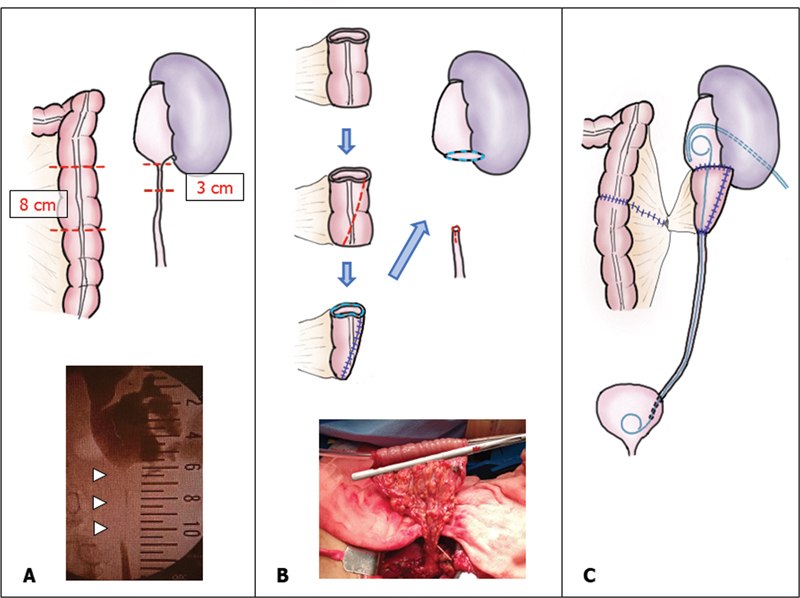
Upper ureteral reconstruction with a tapered segment of descending colon. ( **A** ) Preoperative retrograde pyelography revealed a 5-cm-long stenosis of the ureter ( *white arrowheads* ). ( **B** ) An 8-cm-long descending colon ( **A** ) was isolated, tapered ( *red dotted line* ), sutured in a funnel shape, and then anastomosed to reconstruct the upper ureter. The photograph shows the colon segment being grasped before being tapered and sutured using a catheter as a temporary stent. ( **C** ) The final outcome with a double J stent, percutaneous nephrostomy tube, and retroperitoneal drain.

The presented case is a biracial (Japanese/Caucasian) male, born at 38 weeks of gestation by spontaneous vaginal delivery at a hospital overseas after an uneventful pregnancy. Routine prenatal ultrasonography (US) showed no evidence of hydronephrosis. He was born overseas weighing 2,800 g and grew steadily following standard growth curves overseas until sudden onset of vomiting and abdominal pain at the age of 8 years that was considered psychosomatic. Eventually, he was diagnosed with UPJ obstruction at the age of 11 years (details unknown) and had open pyeloplasty for left UPJ obstruction associated with left hydronephrosis, overseas. His postoperative course was complicated by abdominal pain after removal of a double J stent (DJS) inserted intraoperatively. A DJS was reinserted with resolution of symptoms. Transurethral retrograde pyelography (RP) performed 3 months later at a hospital in Japan during a visit identified obstruction at the ureteropelvic anastomosis (UPA). The Covid-19 pandemic prevented the family from returning overseas and open redo pyeloplasty was performed retroperitoneally elsewhere in Japan, 5 months after the initial operation that was performed overseas. Unfortunately, transurethral RP at the time of postoperative DJS removal identified re-obstruction of the UPA. He was referred for further management of recurrent UPJ obstruction. At presentation when he was almost 13 years old, the RP identified a 5-cm-long stenosis at the UPA and hypoperistalsis of the UPJ and upper ureter ( Fig. 1A ). Dimercaptosuccinic acid (DMSA) scintigraphy revealed almost no difference in split renal function (right/left uptake: 16.6/18.5% [ratio: 47.4:52.6%]). Options for surgical intervention were limited because of the length of the stenosis causing UPJ obstruction, and reconstruction of the upper ureter using the TDC was planned because of the proximity of the descending colon. By this time, 3 months had elapsed since referral and he was 13 years old.

Retroperitoneoscopic repair was planned initially, but it was converted to an open procedure because of extensive adhesions. The ureter was dissected as far as the lower pole of the kidney. The old UPA was exposed and the stenosed segment was excised. Both cut ends of the ureter were thick, fibrosed, and bled easily, appearing macroscopically to be severely inflamed. Some 3 cm of the inflamed lower ureter was also resected ( Fig. 1A ) until the ureter appeared healthier. Because the upper cut end of the old UPA was no more than a fine opening ( Fig. 1B ), it was incised and the incision extended to the renal parenchyma. The mucous membranes of the renal pelvis and calyces also appeared to be inflamed because they were friable and coarse like the cut ends of the ureter, so they were everted to be in direct contact with the TDC at the new anastomosis. After excising the stenosis, there was a 6-cm-long defect requiring tissue interposition.

The peritoneum adjacent to the excised ureter was incised and a length of descending colon was mobilized to the retroperitoneal space. The maximum diameter of the descending colon mobilized was 3 cm. An 8-cm segment of the mobilized colon with intact blood vessels was isolated, tapered, and sutured into a funnel shape with an upper diameter of 2 cm and a lower diameter of 1 cm ( Fig. 1A, B ) around a 14-Fr catheter as a temporary stent. After colocolostomy, the descending colon was returned to the abdominal cavity and the peritoneum closed carefully to prevent vascular compromise. Because the lumen of the lower end of the ureter was also narrow, the antimesenteric side of the ureter was incised until there was healthy, well-preserved tissue that was wide enough for anastomosis to the TDC. A DJS (5 Fr, 26 cm) was placed between the renal pelvis and the bladder via the TDC. The ureter–TDC and calyx–TDC anastomoses were performed with 5–0 absorbable interrupted sutures. A 9-Fr percutaneous nephrostomy tube and a 15-Fr retroperitoneal drain were placed ( Fig. 1C ). The total operative time was 770 minutes.

**Fig. 2:**
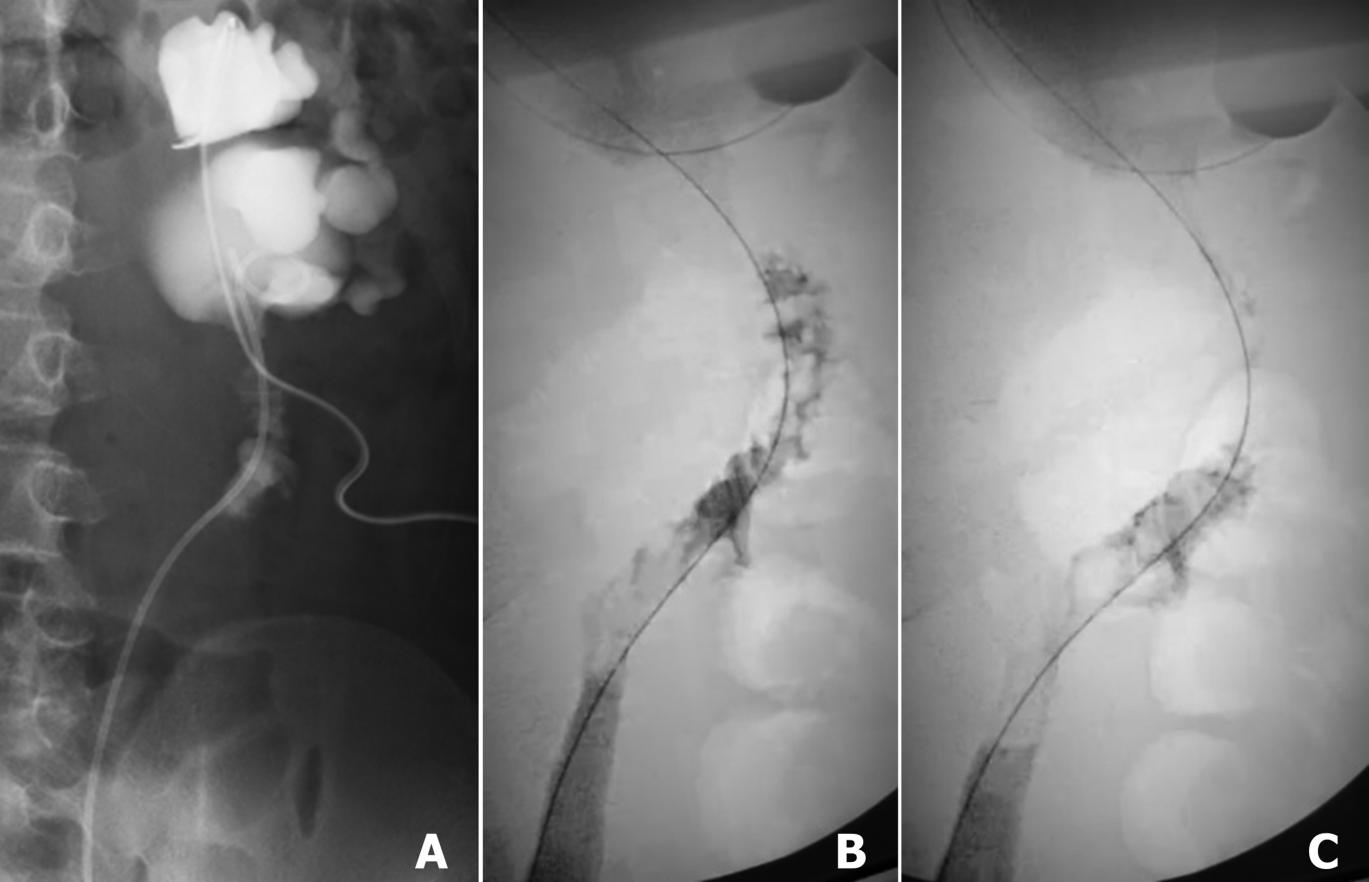
Postoperative urography. Smooth urine flow through the ( **A** ) calyx–tapered segment of descending colon (TDC) anastomosis 13 days postoperatively and through the ( **B,C** ) ureter–TDC anastomosis 49 days postoperatively.

The retroperitoneal drain was left in situ for longer than usual for fear of suture failure at the anastomosis and was removed 12 days postoperatively when drainage decreased. Contrast pyelography via the percutaneous nephrostomy tube the next day showed passage through the calyx–TDC anastomosis but not through the ureter–TDC anastomosis ( Fig. 2A ). Clamping the nephrostomy tube was trialed on postoperative day 14, and gradually extended until it was removed on day 19 without sequelae. The 5-Fr DJS was removed transurethrally on day 49; good passage through the ureter–TDC anastomosis was identified on RP ( Fig. 2B, C ). Diuretic renography performed 2 months postoperatively showed no obstruction on the left side. Currently, he is symptom free after 18 months of follow-up.

## Discussion

The presented case posed problems for reconstructive surgery because of extensive adhesions and long upper ureteral stenosis. An ileal conduit or the appendix accessed intraperitoneally could be considered for primary repair, but a repeat intraperitoneal procedure was essentially contraindicated by the two previous failed pyeloplasties. Because of concern that another extensive intraperitoneal procedure may not proceed well because of severe adhesions with an increased risk of complications such as bowel obstruction, 7 postoperatively, the TDC was chosen specifically as the original strategy was to use a retroperitoneal approach to avoid extensive adhesions expected to be present after failed previous surgery and the descending colon was the closest readily available tissue to the operative field. The TDC was also chosen because it was considered easier than the re-tubularizing transverse colon segments, which, in the presented case, would have required joining at least two segments of the re-tubularized colon in sequence to create the required 6-cm length to bridge the gap in the upper ureter, increasing risks of suture failure and anastomotic leakage because the colon was only 3 cm in diameter. In addition, with the TDC, a ureter can be customized, in this case, into a funnel shape required to connect the larger diameter of the pyelocalyx with the smaller diameter of the ureter, effectively. In fact, the TDC resulted in a continuous neoureter compared with a re-tubularized colon neoureter that would have been comprised of segments.

Because the colon was used to reconstruct the upper ureter, mucus secretion was expected to cause complications such as obstruction or calculus formation. However, in reports of Yang–Monti 6 7 8 cases, when segments of the GIT were used to reconstruct a ureter, the re-tubularization process seemingly disrupted the physiologic function of GIT segments with no complications related to mucus or metabolic consequences of the absorption of substances in urine being identified. In fact, an unexpected advantage of the TDC was efficient antegrade urine flow due to the use of mucosal folds and muscles of the segment of the colon.

The mechanism by which the previous pyeloplasties failed can only be speculated about. Dense fibrous adhesive tissue forming around the UPA, ischemia, foreign body reactions to stents or sutures, missed crossing vessels, kinking of redundant pelvis tissue, or even the use of electrocautery have all been implicated in the etiology of failed pyeloplasty. 9 In the present case, no abnormally crossed vessels were identified and the old UPA was not kinked. Histopathologic analysis was not performed because no surgical specimen was collected from the old UPA; however, there was obvious scarring around the upper ureter. Therefore, the probable cause of the long upper ureteral stenosis and failed pyeloplasties was attributed to scarring of the old UPA.

Contact between the colonic mucosa and urine is a known risk factor for malignant transformation. 10 However, there is evidence that the Yang–Monti technique may not be associated with malignant transformation in the colon 7 and in reports of malignant transformation most patients were older than 50 years. 9 While malignant transformation can be symptomatic with fever of unknown origin, localized discomfort, or abnormal serum biochemistry, routine screening with cystoscopy/colonoscopy every 5 to 10 years or CT colonography every 5 years for patients ≥50 years with GIT segment tissue interposition is recommended. 10 11 For the present case, magnetic resonance imaging may be more appropriate for investigating hematuria or hydronephrosis identified on follow-up US, because it is noninvasive.

## Conclusion

Although other ureteral reconstruction methods using the colon have been reported, this would appear to be the first report in the English language of the TDC being used to create a funnel-shaped segment to reconstruct a long upper ureteric stenosis following two failed pyeloplasties. To date, no complications associated with colonic mucus secretion have been reported. For pediatric surgeons who perform pyeloplasty, this procedure may be a useful option if hydronephrosis recurs and a long upper ureteric stenosis is seen.

## Notes

New Insights & the Importance for a Pediatric Surgeon Long upper ureteral stenosis may require tissue interposition for successful repair. Tapering a segment of the descending colon is simple and seems unaffected by mucus-related complications.
